# A Structure-Based Classification and Analysis of Protein Domain Family Binding Sites and Their Interactions

**DOI:** 10.3390/biology4020327

**Published:** 2015-04-09

**Authors:** Anisah W. Ghoorah, Marie-Dominique Devignes, Seyed Ziaeddin Alborzi, Malika Smaïl-Tabbone, David W. Ritchie

**Affiliations:** 1 Department of Computer Science and Engineering, University of Mauritius, 80837 Reduit, Mauritius; E-Mail: a.ghoorah@uom.ac.mu; 2 CNRS, LORIA, Campus Scientifique, BP 239, 54506 Vandoeuvre-lès-Nancy, France; E-Mail: devignes@loria.fr; 3 Inria Nancy—Grand Est, 54600 Villers-lès-Nancy, France; E-Mail: seyed-ziaeddin.alborzi@inria.fr; 4 University of Lorraine, LORIA, Campus Scientifique, BP 239, 54506 Vandoeuvre-lès-Nancy, France; E-Mail: malika.smail@loria.fr

**Keywords:** protein secondary structure, protein domain, protein binding sites, protein-protein interactions, domain-domain interactions, interaction promiscuity

## Abstract

While the number of solved 3D protein structures continues to grow rapidly, the structural rules that distinguish protein-protein interactions between different structural families are still not clear. Here, we classify and analyse the secondary structural features and promiscuity of a comprehensive non-redundant set of domain family binding sites (DFBSs) and hetero domain-domain interactions (DDIs) extracted from our updated KBDOCK resource. We have partitioned 4001 DFBSs into five classes using their propensities for three types of secondary structural elements (“*α*” for helices, “*β*” for strands, and “*γ*” for irregular structure) and we have analysed how frequently these classes occur in DDIs. Our results show that *β* elements are not highly represented in DFBSs compared to *α* and *γ* elements. At the DDI level, all classes of binding sites tend to preferentially bind to the same class of binding sites and *α*/*β* contacts are significantly disfavored. Very few DFBSs are promiscuous: 80% of them interact with just one Pfam domain. About 50% of our Pfam domains bear only one single-partner DFBS and are therefore monogamous in their interactions with other domains. Conversely, promiscuous Pfam domains bear several DFBSs among which one or two are promiscuous, thereby multiplying the promiscuity of the concerned protein.

## 1. Introduction

Protein-protein interactions (PPIs) are central to many biological processes. At the three-dimensional (3D) structural level, proteins often perform their function by interacting with other proteins to form protein-protein complexes. These complexes may consist of homo-dimers or higher order homo-multimers, or they may involve heteromeric interactions between different protein chains. While homo-interactions are observed relatively often in crystal structures, most processes of biological interest involve hetero interactions, and the corresponding structures are normally much more difficult to determine experimentally and to predict computationally [1]. Consequently, although the number of solved 3D protein structures appears to be growing exponentially [2], there is an equally growing need to be able to classify and analyse the structural repertoire of known hetero PPIs using computational modeling and analysis techniques.

In order to relate the structure and function of different proteins in a systematic way, PPIs are often described in terms of domain-domain interactions (DDIs) because protein domains may often be identified as structural and functional units. Three widely used domain definitions are Pfam [3], SCOP [4], and CATH [5]. Pfam defines domains using multiple sequence alignments in order to identify families of sequences which will often correspond to distinct functional and structural regions. The SCOP and CATH classifications use both sequence and structural similarities to collect and relate protein domains in a hierarchical system of related domain families. However, these two classifications are constructed using different sequence-based and structure-based alignment tools, and they both require the use of considerable human expertise to deal with novel structures which cannot be classified automatically. Thus, they can exhibit important differences at the functional level, and it is becoming increasingly difficult to keep such structural classifications up to date as new protein structures are solved [6]. We therefore choose to work directly with sequence-based Pfam classification, which does not attempt to define a complex structural hierarchy like SCOP and CATH. Several previous studies have shown that proteins often interact via just one or a small number of binding sites [7,8,9], and several authors have studied the biophysical properties of protein binding sites and interfaces. For recent reviews, see e.g., [1,10,11,12]. For example, Keskin *et al.* (2008) [11] summarise the factors which have been examined by several groups in order to characterise protein binding sites. These include, residue conservation, the types of secondary structures present, the shape and surface area of the binding site, the number of water molecules buried on binding, the number of polar and non-polar residues, the number of available hydrogen bonds and salt bridges, and the presence of so-called “hot spot” residues, for example.

In one of the earliest structural studies of PPIs, Janin and Chothia [13] observed that the binding sites of different protease inhibitors and antibody proteins often have similar structural properties. On the other hand, Jones and Thornton [14] found that many interfaces have roughly equal proportions of helix, sheet, and loop residues, with some interfaces containing only one type of secondary structure, but most being mixed. Lo *et al.* [15] found that the size of the recognition site is related to the conformational changes. Xu *et al.* [16] suggest that hydrogen bonds are weaker in the interface, and that salt bridges play a significant role in protein association. Other studies based on alanine scanning mutagenesis have shown that certain hot spot interface residues can contribute a large proportion to the total binding energy [17], and that large amino acids such as tryptophan, arginine, and tyrosine are frequent hot spot residues. Ma *et al.* [18] found that hot spot residues such as tryptophan, and to a lesser extent phenylalanine and methionine, are more structurally conserved in PPI interfaces than in other surface regions, and they proposed that this tendency might be used to help predict the locations of unknown binding sites. Caffrey *et al.* [19] found that the residues at protein interfaces are usually more conserved than other surface residues, particularly in homo-dimers, but they found that such differences are not sufficient to predict interface patches by conservation alone. Guharoy and Chakrabarti [20] reported a comparison between homo-dimeric and hetero-dimeric protein-protein interactions, based on the analysis of secondary structures present at the interface. Interesting trends were observed such as the preference for non regular (neither helix, nor strand) secondary structure in hetero-dimers compared to homo-dimers, and in parallel *versus* perpendicular packing of helices in homo-dimers *versus* hetero-dimers.

Eleven binding motifs were identified visually to capture the prominent architectural features of most of the interfaces. However this study only concerned 122 homo-dimers and 204 hetero-dimers. To our knowledge no updated study has confirmed the relevance of these motifs on a larger scale. Thus, even though some general trends have been observed, it is not clear how best to use this knowledge to differentiate and classify different protein binding sites and interactions. Indeed, we recently argued that there is still no universally accepted definition of what actually constitutes a protein binding site *per se* [9].

In a previous study, we used spatial clustering of hetero DDIs to define the concept of a protein domain family binding site (DFBS). In other words, a DFBS is the abstract representation of all 3D binding-site instances located at the same position on a considered domain family, in all recorded DDIs involving this binding site. As a natural extension of this idea, we then define a domain family interaction (DFI) as an interaction between two DFBSs. Thus a DFI is the abstract representation of all DDI instances that involve the same pair of DFBSs on the two interacting domain families [9]. Although these definitions could cope with any classification of domain instances into domain families, we base our study on the Pfam domain classification for the reasons discussed above. Thus hereafter, protein domain families will be referred to as “Pfam domains”.

Here, we analyse the secondary structural features of DFBSs and DFIs using our KBDOCK collection of 5139 non redundant hetero DDIs corresponding to 3084 DFIs and involving 2153 different Pfam domains and a total of 4001 DFBSs. Indeed, the present work represents one of the largest systematic studies of structural domain interactions to have been described to date and, to our knowledge, the first study to have considered quantitatively the nature of such interactions at the domain family level.

## 2. Methods

### 2.1. The KBDOCK Database

The KBDOCK database has been described previously [9,26]. Briefly, KBDOCK combines information extracted from the Pfam protein domain classification [27] with coordinate data for structural DDIs from the Protein Data Bank (PDB) [28]. The latest version of KBDOCK uses Pfam version 27.0 and a snapshot of the PDB that we took in June 2013. In this study we only deal with hetero DDIs involving two distinct PDB chains. However, many of the DDIs extracted directly from PDB structures are redundant, either because a single crystal structure may contain several symmetry-mates, or because a given complex may have been solved several times under different crystallographic conditions, for example. Therefore, to achieve a robust classification and reliable statistics, KBDOCK eliminates redundant DDIs by applying the NRDB90 program [29] with a threshold of 99% sequence identity to the entire set of sequences built from the concatenation of the two interacting domain sequences in each DDI. It then superposes and spatially clusters the remaining DDIs in order to identify a small number of DFBSs for each Pfam domain [9]. Finally, the DDI instances involving each DFBS are filtered again, this time using a 60% sequence similarity threshold, in order to retain mostly distinct pairs of domains associated with any given DFBS.

For example, the PDB contains 11 hetero DDIs for the Kunitz_legume domain (Pfam accession No. PF00197) which KBDOCK spatially clusters to identify 4 DFBSs on this domain. For each DFBS, the DDIs are filtered using a 60% sequence identity threshold, which gives a total of 5 non-redundant hetero DDIs corresponding in this case to 5 DFIs. More specifically, one DFBS of Kunitz_legume binds both Trypsin (PF00089) and Peptidase S8 (PF00082), another binds a different DFBS on Trypsin, the third binds Alpha amylase (PF00128), and the fourth binds Thioredoxin (PF00085).

Overall, when considering only hetero interactions, the above filtering and clustering procedures give a total of 4001 Pfam DFBSs located on 2153 different Pfam domains or families, and involved in a total of 5139 non redundant DDIs. As two or more non redundant DDIs can still correspond to the binding between the same two Pfam domains at the same binding sites, the 5139 non redundant hetero DDIs have been mapped to a total of 3084 distinct DFIs. The KBDOCK web server is available at http://kbdock.loria.fr. This provides an easy-to-use public interface to explore and analyse domain family interactions and their binding sites and to propose protein docking templates. In order to carry out the analyses presented here, several specialised KBDOCK queries were implemented in Prolog. A full dump of the database is also available from the above web site.

### 2.2. Structural Annotation of DFBSs

We use the DSSP program [21] to annotate domain and DFBS residues with secondary structural information. DSSP defines eight types of SSE, namely, *α*-helix (H), 3/10-helix (G), *π*-helix (I), residue in isolated *β*-bridge (B), extended strand (E), hydrogen bonded turn (T), bend (S), and loop/irregular (L). However, because several of these types are broadly quite similar, and because only a few instances of turns and bends were found in the KBDOCK database, the eight DSSP types were grouped into three main SSE types which we denote here as *α* (H, G and I), *β* (B and E), and *γ* (T, S, and L).

Given a binding site DFBS*_f,b_* involving Pfam domain *f* and binding site *b*, let *D_f,b_* represent the set of non redundant DDIs that involve this binding site. In fact, 596 of the 4001 DFBSs studied here belong to more than one DDI instance in our set of 5139 non-redundant DDIs. Equation (1) describes how we calculate the three SSE propensities *P_f,b_*(*s*) for *s* ∈ {*α*, *β*, *γ*}.

(1) $$P_{f,b}\left(s\right)=\frac{1}{\left|D_{f,b}\right|}\sum_{m\in D_{f,b}}\frac{N_{m,b}^{s}}{N_{m,b}^{\alpha }+N_{m,b}^{\beta }+N_{m,b}^{\gamma }},$$

where $N_{m,b}^{s}$ is the count of interaction residues of class *s* at binding site *b* of member *m* ∈ *D_f,b_*. Each SSE propensity value calculated in this way is automatically normalised to fall within the range [0, 1].

Similarly, Equation (2) shows how we calculate the SSE propensity of a whole domain surface, *Q_f,b_*(*s*).

(2) $$Q_{f,b}\left(s\right)=\frac{1}{\left|D_{f,b}\right|}\sum_{m\in D_{f,b}}\frac{N_{m}^{s}}{N_{m}^{\alpha }+N_{m}^{\beta }+N_{m}^{\gamma }}.$$

This calculation uses the same set of DDIs as above but $N_{m}^{s}$ now represents the count of surface residues of class *s* on the whole domain surface of member *m* ∈ *D_f,b_*.

It can be noted that by construction we have

*P_f,b_*(*α*) + *P_f,b_*(*β*) + *P_f,b_*(*γ*) = 1 (3)

and

*Q_f,b_*(*α*) + *Q_f,b_*(*β*) + *Q_f,b_*(*γ*) = 1. (4)

## 3. Results

### 3.1. Classifying and Analysing DFBSs

The secondary structural features of DFBSs and domain surfaces were estimated after annotating each amino acid residue with the DSSP program [21]. The eight original DSSP types were grouped into three secondary structure element (SSE) classes, denoted here as *α*, *β* and *γ*. The three SSE propensities were then calculated as described in the Methods section. We initially attempted to define SSE classes automatically using various clustering methods (hierarchical, expectation maximization, and density clustering). However, no well separated clusters ever appeared in the data and the calculated log-likelihood values were not significant. Furthermore, data visualization suggested a quasi-continuous distribution of the data over the three SSE classes.

**Figure 1:**
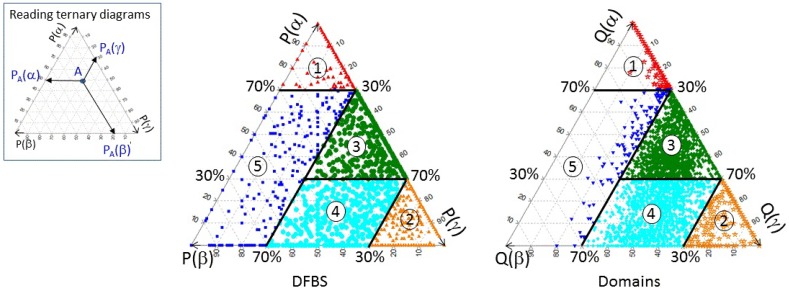
Ternary diagrams representing the overall distribution of the 4001 DFBSs (left diagram) and of the corresponding 4001 domain surfaces (right diagram) according to their per cent SSE propensities, *P*(*s*) and *Q*(*s*), respectively. Dots are coloured as red: class 1; orange: class 2; green: class 3; cyan: class 4; blue: class 5. Heavy lines indicate our chosen SSE class boundaries. The box on the left illustrates how to read the diagram.

Consequently, we decided to analyse the overall distribution of the three SSE propensities over DFBSs and domain surfaces using ternary diagrams (http://mypage.iu.edu/tthomps/programs/html/tnttriplot.htm). In order to do this, the three SSE propensities were plotted in percent units on the ternary diagram, for each binding site and for each domain surface, as shown in Figure 1. The inset box in this Figure shows how to read such ternary diagrams. It may be recalled that, by construction, the sum of the three coordinates is always equal to 100%.

As can be seen from the ternary diagrams in Figure 1, both DFBSs and whole domain surfaces show a rather continuous distribution of the three SSE propensities. Thus, it is not surprising that our attempt to cluster automatically DFBSs and domain surfaces was not successful. On the other hand, visual inspection of this Figure led us to partition the data into 5 SSE classes delineated on the figure by the heavy lines representing *P*(*α*) = 70% and *P*(*α*) = 30% on the one hand, and *P*(*γ*) = 70% and *P*(*γ*) = 30% on the other hand. Numerical details of the resulting 5 SSE classes are given in Table 1. Although this choice is somewhat arbitrary, it can be seen from Figure 1 that it provides a well-balanced partitioning of the distribution of SSE propensities in both DFBSs and whole domain surfaces.

**Table 1:** SSE class definitions and distribution of DFBSs according to the selected ternary diagram contour thresholds.

Definition	Class No.	No. DFBSs	No. Domains
*P*(*α*) ≥ 70%	1	835	504
*P*(*γ*) ≥ 70%	2	996	249
30% ≤ *P*(*γ*) < 70% AND 30% ≤ *P*(*α*) < 70%	3	1117	1805
30% ≤ *P*(*γ*) < 70% AND *P*(*α*) < 30%	4	784	1319
*P*(*γ*) < 30% AND *P*(*α*) < 70%	5	269	124

The means and standard deviations (SDs) of the SSE propensities calculated for the 5 classes for both DFBSs and domain surfaces are shown in Table 2. Inspecting the percentages in this table, naturally leads us to refer to class 1 as “*α*-rich”, and class 2 as “*γ*-rich”, because *α* and *γ* SSEs clearly predominate in these two classes, respectively. Similarly, it is natural to name class 3 as “*α* + *γ*”, class 4 as “*β* + *γ*”, and class 5 as “*α* + *β* + *γ*”, because this last class always has a significant proportion of each SSE type. It is worth noting that because the number of DFBSs and domain surfaces having *P*(*β*) ≥ 70% (99 and 3, respectively) is too low to be considered even as a subclass of the poorly populated *α* + *β* + *γ* class (Table 1), our classification does not have a specific “*β*-rich” class. Nonetheless, it is interesting to note that DFBSs in the *α* + *β* + *γ* class contain mainly *β* SSEs (53.1%), whereas whole domain surfaces in this class contain mainly *α* SSEs (also 53.1%).

**Table 2:** The Mean and SD (percent units) of the SSE propensities for the five defined DFBS classes.

Class No.	1	2	3	4	5
Class Name	*α*-rich	*γ*-rich	*α* + *γ*	*β* + *γ*	*α* + *β* + *γ*
DFBS					
*P*(*α*)	86.6 ± 10.7	7.3 ± 9.8	48.5 ± 11.5	9.3 ± 10.0	29.3 ± 24.7
*P*(*β*)	0.5 ± 2.8	5.5 ± 8.7	3.9 ± 7.6	39.1 ± 15.1	53.1 ± 27.5
*P*(*γ*)	12.9 ± 10.5	87.2 ± 11.2	47.6 ± 10.9	51.7 ± 10.8	17.6 ± 9.5
Domain Surface					
*Q*(*α*)	79.7 ± 7.6	9.6 ± 9.9	46.7 ± 11.2	14.7 ± 9.6	53.1 ± 12.8
*Q*(*β*)	0.2 ± 0.9	0.9 ± 8.0	9.7 ± 9.0	33.0 ± 12.0	21.0 ± 12.5
*Q*(*γ*)	20.1 ± 7.5	82.4 ± 10.4	43.6 ± 8.3	52.3 ± 8.6	26.0 ± 2.9

### 3.2. Are Binding Site Surfaces Special?

Because each DFBS constitutes part of a domain surface, we first wished to investigate whether the SSE propensities of a DFBS are related to the overall SSE propensities of its complete domain’s accessible surface. Table 3 shows the percentages of DFBSs belonging to each of the five SSE classes for each class of domain surface. Comparing these percentages with the last row of DFBS frequencies averaged over all domains reveals significant differences, suggesting that the SSE composition of DFBSs strongly depends on the overall SSE composition of their domains. Indeed, particularly high values occur on the main diagonal of Table 3, where DFBSs have the same class as their domain surface. This is especially the case for the *α*-rich and *γ*-rich classes (72.4% and 80.3%, respectively).

**Figure 2:**
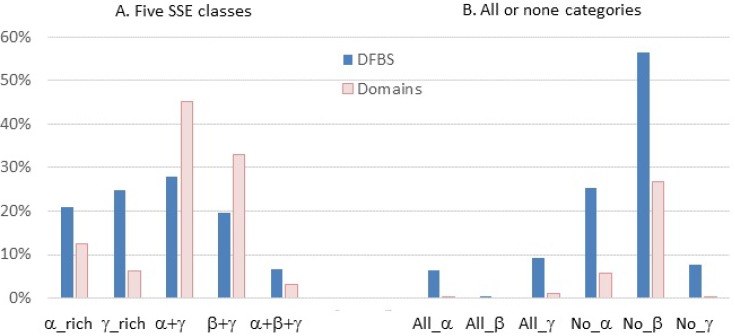
Histogram of the distribution of DFBSs and domain surfaces over the five SSE classes (**left**) and the “all” or “none” SSE categories (**right**).

We also counted the numbers of DFBSs and domains having certain “extreme” SSE compositions such as “all-*α*”, “all-*β*”, “all-*γ*” (*i.e.*, points at the three vertices of the ternary diagram), and “no-*α*”, “no-*β*”, “no-*γ*” (*i.e.*, points on the ternary diagram axes). The histogram in Figure 2 shows how the distribution of DFBSs and domain surfaces vary over the five SSE classes (A) and in the extreme “all” or “none” categories (B). It can be seen that the *α*-rich, *γ*-rich, and *α*+*β*+*γ* classes are more frequent among DFBSs than among whole domain surfaces, especially for the *γ*-rich class, whereas *α* + *γ* and *β* + *γ* are more frequent in whole domains than in DFBSs. It can also be seen that it is much more frequent for the “extreme” SSE compositions to be observed in DFBSs than in whole domain surfaces. More precisely, 16.3% of DFBSs fall within an “all” category, compared to only 1.3% of domain surfaces, and 89.2% of DFBSs belong to a “none” category compared to only 39.5% of domain surfaces. Within the particular “all” and “none” categories, the partition between *α*, *β*, and *γ* is roughly the same for both DFBSs and domain surfaces, with a majority of “all-*γ*” and very low “all-*β*” on the one hand (with just 18 DFBSs, or 0.4%, and no domain surfaces), and with a majority of “no-*β*” and very low “no-*γ*” on the other hand. More than half of the DFBSs considered in this study lack any *β*-strand residues (56.4%).

**Table 3:** Percent probabilities of observing a particular class of DFBSs with respect to the SSE class of the whole of the corresponding domain’s surface.

Domain Surface	DFBS Class				
	*α*-rich	*γ*-rich	*α* + *γ*	*β* + *γ*	*α* + *β* + *γ*
*α*-rich	72.4	4.2	23.0	0.0	0.4
*γ*-rich	1.6	80.3	7.2	10.4	0.4
*α* + *γ*	22.0	19.4	42.7	9.8	6.1
*β* + *γ*	2.7	31.8	13.7	43.1	8.7
*α* + *β* + *γ*	26.6	4.8	25.0	10.5	33.1
All domains	20.9	24.9	27.9	19.6	6.7

Overall, these results confirm the previous finding of Guharoy and Chakrabarti [20] that irregular secondary structure (here called *γ*) are very common in protein-protein binding sites, especially in hetero-complexes. In addition, our results show that *β*-type SSEs are generally under-represented both on whole domain surfaces and in binding sites, going to a complete absence of *β* elements in more than 50% of DFBSs. Importantly, our analysis also shows that the SSE composition of a DFBS is largely determined by the overall SSE composition of the domain to which it belongs (Table 3).

### 3.3. Do DFIs have SSE Pairing Preferences?

In order to investigate whether particular pair-wise combinations of SSE classes might exist at domain-domain interfaces, we calculated the numbers of DFIs found for each pair of DFBS SSE class (Table 4). We verified that this distribution is significantly different from the one expected from a random association of any two DFBS SSE classes. To do this, we first calculated the expected percent probabilities of each SSE class pair, assuming that binding occurs randomly. We therefore used the product of the percentages of the corresponding DFBS SSE classes (see values in Table 3, last row). For example the expected probability for the (*α*-rich, *α*-rich) DFBS pair is equal to 20.9%^2^ = 4.4%, and the expected probability for the (*α*-rich, *γ*-rich) pair is equal to 2 *×* 20.9% *×* 24.9% = 10.4% (the percentage product gets doubled because the pair can be either (*α*-rich, *γ*-rich) or (*γ*-rich, *α*-rich)). These random pair-wise probabilities were multiplied by the total number of DFIs in our dataset (3084) to obtain the numbers of expected DFIs for random binding (as shown in parentheses in Table 4).

**Table 4:** Numbers and percentages of observed DFIs for all pairs of DFBS SSE classes in our dataset of 3084 DFIs. In parentheses is the number of DFIs expected if the binding occurs randomly between any two SSE classes of DFBSs. Statistical testing identifies percentages that are significantly higher (bold) or lower (underlined) than the percentages expected from random binding.

	*α*-rich	*γ*-rich	*α* + *γ*	*β* + *γ*	*α* + *β* + *γ*
*α*-rich	**227 (124)**	177 (320)	334 (359)	137 (252)	40 (87)
	***7.4%***	*5.7%*	*10.8%*	*4.4%*	*1.3%*
*γ*-rich		**251 (191)**	327 (429)	**356 (301)**	84 (103)
		***8.1%***	*10.6%*	*** 11.5%***	*2.7%*
*α* + *γ*			**333 (240)**	*72 (337)*	**307 (116)**
			***10.8%***	*2.3%*	***10.0%***
*β* + *γ*				**312 (118)**	97 (81)
				***10.1%***	*3.1%*
*α* + *β* + *γ*					**30 (14)**
					***1.0%***

Thanks to our large dataset, all numbers are greater than 5, and we can use a *χ*^2^ test to assess the difference between the two distributions. The result obtained indicates that the observed distribution of DFIs over the 15 possible SSE class pairs is significantly different from the one expected from random binding (*p* < 0.001). This represents a strong confirmation, on a large dataset, of previous hypotheses that certain SSE classes of binding sites favorably or unfavorably interact with other SSE classes. More precisely, individual comparisons for each type of SSE class pair were performed using a *z*-test. For twelve out of the fifteen types of SSE class pairs, the observed numbers were found to be significantly higher or lower than the random ones (*p* < 0.001). The observed numbers were converted into percentages values and reported in Table 4 with bold characters marking significantly higher percentages and underlined characters marking significantly lower percentages. Not significantly different percentages correspond to the absence of any favorable or unfavorable preference for three SSE class pairs: (*α*-rich, *α* + *γ*), (*γ*-rich, *α* + *β* + *γ*) and (*β* + *γ*, *α* + *β* + *γ*).

It can be seen from Table 4 that all values on the diagonal are higher than random showing that the binding between the same two SSE classes of DFBSs is favored. This is also the case for the binding between *γ*-rich and *β* + *γ* classes of DFBSs and between *α* + *γ* and *α* + *β* + *γ* classes of DFBSs. Other pairs show lower percentages than random. The most striking pair corresponds to the binding between *α* + *γ* and *β* + *γ* classes (72 observed instead of 337 expected) suggesting it is physically difficult to form interfaces between binding sites containing *α*-helices on one hand and *β*-strand on the other hand. The lower percentages observed for the (*α*-rich, *β* + *γ*) and (*α*-rich, *α* + *β* + *γ*) pairs confirm this statement.

**Figure 3:**
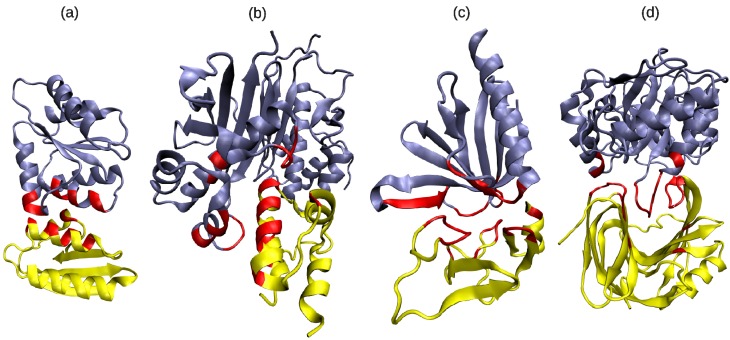
Examples of DDIs involving various SSE class pairs: (**a**) *α*-rich with *α*-rich (PDB code 1VRC, chains A, C); (**b**) *α*-rich with *α* + *γ* (PDB code 2CG5, chains A, B); (**c**) *α* + *β* + *γ* with *α* + *β* + *γ* (PDB code 2YIB, chains F, B); (**d**) *γ*-rich with *β* + *γ* (PDB code 1TE1, chains A, B). Binding site SSEs are shown in red. This figure was drawn using the VMD program (http://www.ks.uiuc.edu/Research/vmd/).

Figure 3 shows some examples of protein complexes with particular SSE class pairs at the domain interface.

### 3.4. Are Multi-Partner Binding Sites Special?

Because our set of DDI is grouped by Pfam domains and DFBSs, it is possible to classify DFBSs according to their number of distinct Pfam partners. We therefore wished to ask whether there are any particular differences between DFBSs having just one Pfam interaction partner, and those having two or more Pfam partners. Table 5 shows the distribution of DFBSs in the five SSE classes depending on their number of Pfam partners. The total number of DFBSs capable of binding to 1, 2, 3 or more than 3 partners is indicated in the first column. It can be seen that 81.1% (3245 out of 4001) DFBSs only interact with one partner, and that very few (3.5%) interact with more than 3 distinct Pfam partners. For these DFBSs the number of partners ranges from 4 to 49 (average 6.7, median 5), excluding an outlier DFBS having 142 distinct Pfam partners (belonging to the Immunoglobulin V-set domain PF07686).

**Table 5:** Distribution of DFBSs in the five SSE classes (percent units) depending on the number of Pfam partners. Significant differences from the global distribution (last row) are indicated with underlined or bold characters for lower or higher percentages, respectively.

DFBSs	No. Partners	*α*-rich	*γ*-rich	*α* + *γ*	*β* + *γ*	*α* + *β* + *γ*
3245	1	21.6	24.4	28.0	18.8	7.2
499	2	18.4	28.7	28.5	19.8	4.6
119	3	21.8	28.6	22.7	21.9	5.0
138	>3	10.9	19.6	29.7	**36.2**	3.6
4001	Any	20.9	24.9	27.9	19.6	6.7

Statistical comparison of the distribution of DFBSs over the five SSE classes in each row with the global DFBS distribution (last row) reveals that this distribution is significantly different (*χ*^2^ test, *p* < 0.001) only when the number of partners is greater than 3. In this row, individual comparisons for each SSE class (*z*-test) yields a significant difference only for the *α*-rich (lower percentage, *p* < 0.01) and *β* + *γ* (higher percentage, *p* < 0.001) classes. This observation suggests that, to accommodate large numbers of partners, the binding sites tend to involve decreased *α* and increased *β* secondary structure.

Finally, we used DSSP to calculate the solvent accessible surface (SAS) of each DFBS (this calculation assumes that each binding site contributes equally to the buried SAS at a DDI interface). Table 6 shows the average surface areas and residue numbers of DFBSs depending on their number of Pfam partners. The table suggests that DFBSs having more than one interaction partner at the family level tend to have slightly smaller binding site surface areas than single-family DFBSs (two-sample *z*-test, *p* < 0.01). A similar trends towards a decrease in the number of residues involved at the interface is observed when compared with single-partner DFBSs (*p* < 0.01 for two-partners DFBSs and *p* < 0.05 for three partners, not significant for more than three partners).

**Table 6:** Average DFBS surface areas calculated with respect to their number of Pfam domain partners.

DFBSs	No. Partners	Average SAS (Å^2^)	No. Residues
3245	1	678 ± 496	12 ± 9
499	2	579 ± 429	10 ± 8
119	3	554 ± 482	10 ± 9
138	≥4	578 ± 334	11 ± 7

### 3.5. Distribution of DFBSs on Multi-Partner Pfam Domains

**Figure 4:**
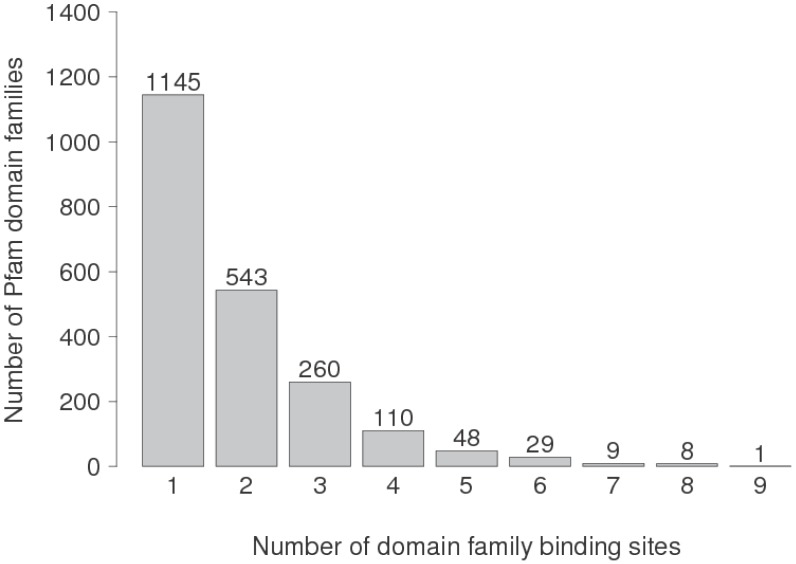
The distribution of Pfam domains depending on their number of DFBSs per domain.

KBDOCK allows to monitor multiple binding capacities at the levels of both individual DFBSs and Pfam domains. In particular, it is quite natural to think that Pfam domains having the highest number of Pfam partners also have the highest number of DFBSs. This led us to analyse the distribution of Pfam domains depending on the number of DFBSs per domain, as shown in Figure 4. Interestingly, 53.2% (1145/2153) of Pfam domains harbour just one DFBS, of which 91% (1041/1145) are single-partner DFBSs. Thus, almost half of Pfam domains in our dataset are primarily monogamous in their physical relationships with other domains.

In fact only 9.5% (205/2153) of Pfam domains harbour four or more DFBSs, with a maximum of nine for Actin domain (PF00022). The final promiscuity of these domains then depends on the promiscuity of each harboured DFBS. More concretely, Table 7 lists the binding multiplicities of the nine Pfam domains having the highest numbers of DFBSs per domain (excluding the outlier case of Immunoglobulin V-set domain already mentioned above). As might be expected, most of these domains such as Ras, PKinase, Ubiquitin, and C1-Set are central to essential cellular processes such as regulation, signaling, and the immune system, for example.

**Table 7:** The numbers of distinct Pfam domain partners for the nine Pfam domains having the greatest numbers of DFBSs.

Pfam Domain	DFBSs	Partners per DFBS									Total Partners
Ubiquitin-conjugating enzyme	6	2	4	11	5	3	1				19
Ubiquitin	6	3	29	3	1	1	6				36
Ras	7	49	3	3	1	3	1	1			56
Trypsin	7	26	7	13	7	4	4	2			44
Immunoglobulin C1-set	7	5	5	14	8	2	1	8			33
Protein kinase domain	8	16	18	6	1	1	1	3	1		39
Class I histocompatibility antigen	8	1	5	5	2	1	1	1	1		10
RNA polymerase Rpb1	8	1	3	2	1	2	1	1	1		10
Actin	9	3	10	4	2	2	1	1	1	1	21

It is interesting to see that the Actin domain currently has the highest number of DFBSs but not the highest number of Pfam partners, which is held by the Ras domain (56 *versus* 21). This is consistent with the promiscuity of individual DFBSs on these two domains, which is up to 49 for Ras, but only 10 for actin. Thus, promiscuity of DFBSs can strongly expand the promiscuity of Pfam domains, especially for domains that are part of hub proteins. Kinase domains, for example, are frequently observed in PPI network hubs, with some 400 hubs having some kind of kinase activity [22]. It can be seen in Table 7 that the Pfam protein kinase domain (PF00069) harbours 8 DFBSs of which two can bind to 16 and 18 different Pfam partners, resulting in a total number of 39 different Pfam partners.

**Figure 5:**
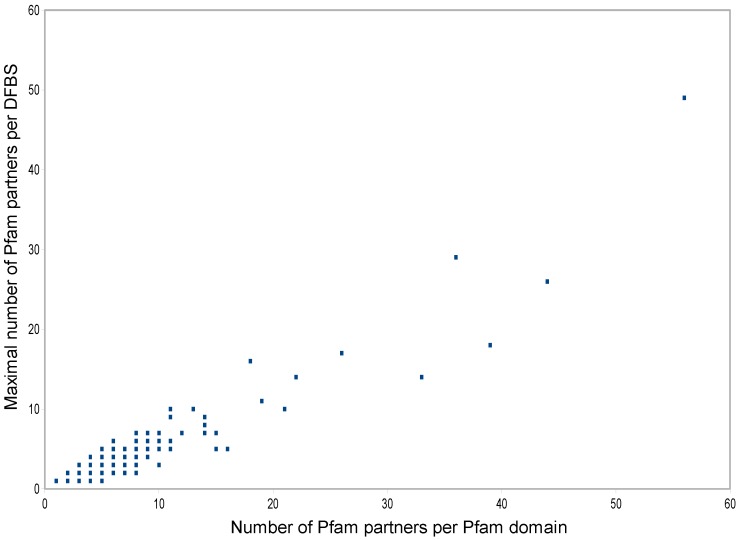
Scatter plot of Pfam domains according to their number of partners (*x* coordinate) and the maximal number of partners per DFBS among the DFBSs they harbour (*y* coordinate). For low values, one dot can represent more than one Pfam domain.

We further analysed the correlation between promiscuity at the DFBS and Pfam domain levels as shown in Figure 5. Individual Pfam domains were plotted using the maximal number of Pfam partners per DFBS found among the DFBSs they harbour (*y* coordinate) *versus* their total number of partners at the Pfam domain level (*x* coordinate). A clear correlation is visible between the two variables, showing that to ensure very high promiscuity, Pfam domains preferentially harbour DFBSs with high promiscuity.

## 4. Discussion

There is considerable interest in understanding how experimentally observed PPI networks can be explained in terms of 3D structural interactions [22,23]. However, it is difficult to rationalise or consolidate the results from different groups because different investigators often use different terminologies to describe different types of interaction (e.g., homo *versus* hetero, transient *versus* permanent, obligate *versus* non-obligate, biological *versus* non-biological contact, date hub *versus* party hub, sociable *versus* non-sociable, “singlish” interface *versus* multi-interface). Such a variety of language highlights the need for a well-defined terminology and methodology with which to describe, classify, and analyse the structural nature of PPIs and protein binding sites.

Several previous studies of structural PPIs have identified potentially interesting relationships between the shapes and physical properties of protein-protein interfaces, and most such studies have suggested that it might be possible to use such properties predictively. However, these earlier studies of PPIs have been somewhat limited by the relatively small numbers of hetero protein-protein complexes available, and by the problem of how to select a suitable sub-set of protein binding sites to work with. Because KBDOCK is built from a complete and recent (June 2013) snapshot of the PDB and the latest version of Pfam (v27.0), our SSE-based classification of binding sites may be considered as the most comprehensive one to date. Indeed, we believe the work described here represents the largest systematic and non-redundant study of protein domain interactions to have been described to date. Furthermore, we believe that the very large coverage and lack of redundancy of our dataset make our classification quite reliable compared to earlier studies which used sub-sets of the PDB. This analysis was possible thanks to the well-characterized and non-redundant set of binding sites that we identified and stored previously in KBDOCK using our spatial clustering algorithm [9]. In this work, we have used ternary diagram representations to show that the SSE composition of DFBSs and whole domain surfaces has a rather continuous distribution. The continuous nature of the SSE coordinates means that it is difficult, if not impossible, to cluster DFBSs automatically into well-defined groups. Nonetheless, we used visual inspection of the ternary diagrams to partition the ternary SSE space into five structural classes using simple rules to define different combinations of the three main SSE types. This partitioning of SSE space provided a practical and objective basis with which to describe and compare different combinations of SSEs in a way that reflects well the observed domain binding-site SSE propensities.

As well as supporting previous findings regarding the preference for *γ*-rich features in binding sites, our results also show that there seems to be nothing particularly special about the SSE composition of a DFBS when compared to the rest of its domain surface. From a global point of view however, our results also show that “extreme” SSE compositions are much more common in DFBSs than in whole domains. More than 25% DFBSs in our dataset completely lack *α* helices, and more than 50% completely lack *β*-strand residues.

Furthermore, our results show that there exist certain non-random pairing preferences between the different SSE classes. In particular, interactions involving the same two SSE classes are more frequent than expected from random binding. Conversely, binding of an *α*-rich or *α* + *γ* DFBS to a *β* + *γ* DFBS is significantly disfavored. Knowledge of these secondary structure pairing propensities could be useful for the prediction of unknown DDIs, especially if combined with other physical properties [10].

Additionally, we find that just over 80% of DFBSs in our dataset interact with just one type of Pfam domain, and only around 3.5% of them interact with more than three different Pfam domains. Our SSE propensity analysis indicates that DFBSs with three or more distinct Pfam partners have a lower probability of being classified as *α*-rich and a higher probability of being classified as *β* + *γ* compared to the overall frequency of these SSE classes. This contradicts somewhat the results of Keskin and Nussinov [8] that multi-partner interfaces preferentially consist of *α* helices, but their study performed in 2007 involved fewer DDIs than ours. We also see a tendency for DFBSs having more than one Pfam interaction partner to have slightly smaller binding site surface areas than single-partner DFBSs. This confirms a previous observation by Keskin *et* *al.* [11] on a larger dataset, who already commented that with a large interface, it would be more difficult to bind to various complementary sites.

More than 50% of Pfam domains in our dataset harbour only one DFBS and 91% of these DFBSs are single-partner. Thus almost half of Pfam domains are monogamous in their physical relationships with other domains, and this finding could have considerable implications for the interpretation of large-scale PPI networks and for drug targeting. Conversely, the “promiscuous” DFBSs are mostly found in domains containing more than one DFBS. In particular the 9.5% of Pfam domains harbouring four or more DFBSs all possess at least one multi-partner DFBS. This suggests there exists a kind of “two-level multiplication” in the number of different partners for these domains. This partner multiplicity could be further enhanced when multi-partner domains assemble in multi-domain architectures. The resulting combinatorial expansion of partners could explain the structural basis for the observed promiscuity of some hub proteins. It has been proposed that the presence of hub proteins in a PPI network is a natural consequence of a “scale-free” network, or one which follows a “power law” distribution in the degree of its network nodes [24]. Another study states that PPI network topology can be either scale-free or have a normal distribution, according to the number of interacting domain pairs [25]. Thus it could be interesting to try to construct and analyse PPI networks for selected biological pathways using only non-redundant structural DDIs from KBDOCK, and to compare their topologies at both the DDI and PPI levels. We are planning future analyses to verify whether the small set of multi-partner DFBSs and their corresponding domains are actually involved in the architectures of hub proteins.

## 5. Conclusions

Our structural classification of DFBSs provides a useful way to classify and analyse the secondary structure propensities of DDIs, and it highlights some SSE pairing preferences which might be useful for the prediction of unknown DDIs. We have used this classification to analyse the structural interactions of a large set of 4001 domain family binding sites located on 2153 Pfam domain families, and involving 5139 non redundant DDIs. Our results show that many DDIs involve *γ*-rich features and that interactions involving *α*/*β* contacts are disfavored. We find that the SSE character of multi-partner binding sites can significantly differ from single-partner binding sites when the number of partners exceeds three. Moreover, multi-partner binding sites are found to have significantly smaller surface areas than single-partner binding sites. Our analysis successfully identifies known hub proteins as containing domains that carry several binding sites among which at least one multi-partner binding site. Nevertheless, we also find that about half of the domains in our study are primarily monogamous in their hetero relationships with other domains, and this finding could have considerable implications for the interpretation of large-scale PPI networks and for drug targeting. We believe it could be very interesting to use the domain family interactions in KBDOCK to reconstruct and analyse PPI networks at a structural level and to investigate in more detail the 3D architectures of hub proteins and the topologies of structural DDI networks.
